# Inflammation and decreased cardiovagal modulation are linked to stress and depression at 36th week of pregnancy in gestational diabetes mellitus

**DOI:** 10.1038/s41598-023-37387-4

**Published:** 2023-06-26

**Authors:** Manoharan Renugasundari, Gopal Krushna Pal, Latha Chaturvedula, Nivedita Nanda, K. T. Harichandrakumar, Thiyagarajan Durgadevi

**Affiliations:** 1 Department of Physiology, JIPMER, Puducherry, India; 2 All India Institute of Medical Sciences (AIIMS), Patna, Bihar India; 3 Department of Obstetrics and Gynecology, JIPMER, Puducherry, India; 4 Department of Biochemistry, JIPMER, Puducherry, India; 5 Department of Biostatistics, JIPMER, Puducherry, India

**Keywords:** Physiology, Psychology, Biomarkers, Endocrinology, Health care

## Abstract

Stress and depression have been reported in gestational diabetes mellitus (GDM). Though inflammation and oxidative stress are associated with depression, there are no reports of link of cardiometabolic risks (CMR) to stress and depression in GDM. Normal pregnant women (control group, n = 164) and women with GDM (study group, n = 176) at 36th week of gestation were recruited for the study. Blood pressure (BP), body composition, heart rate variability (HRV), glycated hemoglobin (HbA1C), markers of insulin resistance, oxidative stress, inflammation and endothelial dysfunction, were assessed. Perceived stress score (PSS), quality of life (QoL) scale, Indian diabetic risk score (IDRS) and Edinburg postnatal depression score (EPDS) were assessed. Association of potential contributors to PSS and EDPS were assessed by correlation and regression analyses. There was significant increase in PSS, EPDS, IDRS scores, HbA1C, malondialdehyde (MDA) (oxidative stress marker) and high-sensitive C-reactive protein and interleukin-6 (inflammatory markers), and significant decrease in total power (TP) of HRV (marker of cardiovagal modulation), QoL and nitric oxide (endothelial dysfunction marker) in study group compared to control group. Though many cardiometabolic risk parameters were correlated with PSS and EPDS, the significant independent association was observed for TP, HbA1C, MDA and interleukin-6. However, interleukin-6 had maximum contribution to PSS (β = 0.550, *p* < 0.001) and EPDS (β = 0.393, *p* < 0.001) as demonstrated by multiple regression analysis. Inflammation, oxidative stress, glycation status and decreased cardiovagal modulation are associated with stress and depression at 36th week of gestation in GDM.

## Introduction

Gestational diabetes mellitus (GDM) is defined as any degree of glucose intolerance with the onset or first recognition during the present pregnancy at 24–28 weeks of gestation^1^. In the recent past, there is an increased incidence of diabetes during pregnancy with almost 21 million births (16.2%) affected due to hyperglycemia^2^. Prevalence of GDM in India is 18.9%, ranging between 3.8% to 41% in various parts of the country^3^. The major maternal and neonatal adverse effects of GDM include increased risk of preterm delivery, pre-eclampsia, caesarean section delivery, development of type 2 diabetes mellitus (T2DM) post-delivery, fetal macrosomia, neonatal hypoglycemia, neonatal respiratory distress, and childhood obesity and insulin resistance, followed by impaired glucose tolerance and T2DM later in life^4,5^. Women with history of GDM have a sevenfold increased risk of developing T2DM, with 20–70% risk in the first decade after delivery and GDM increases the future cardiovascular disease (CVD) risk by twofold^6^. It is important to note that studies have consistently shown that women with a history of GDM have a higher prevalence of cardiometabolic risk factors, such as dyslipidemia, hypertension, obesity, and metabolic syndrome, compared to their peers. Additionally, by three months postpartum, this adverse cardiovascular risk factor profile is evident^6^.

Depression during pregnancy has been reported to adversely affect women and their children^7,8^. Anxiety, psychological stress and depression are associated with GDM^9–12^. Psychological stress and depression in GDM severely affect the maternal and fetal outcomes^13,14^. Also, it has been reported that the prenatal depression in GDM is linked to post-partum depression for a longer duration and adverse cardiovascular (CV) consequences^15,16^. Recently we have reported the decreased heart rate variability (HRV) and cardiovagal modulation associated with depression in women during antenatal period, which exposes them to CV risks^17^. However, till date the mechanism that causes CV risks in GDM have not been well studied and the association of CV risks to mental illness in GDM has not been reported. It is important for healthcare workers to know the relationship between stress and depression with fetomaternal outcomes, and if the depression-associated problems can be prevented in the perinatal period. In a recently conducted pilot study at 36th week of gestation in 15 women having GDM, we observed that cardiometabolic risks, adverse fetomaternal outcomes and poor psychophysical health were improved by practice of short course of yoga^18^. But, in this study due to less sample size, we could not assess the enormity of cardiometabolic risks, the mechanisms that could lead to development of these risks and the link of depression to the risks and the maternal–fetal outcomes.

Inflammation has long been proposed as a pathophysiologic mechanism of depression^19^. There is accumulating evidence that anti-inflammatory interventions could be promising antidepressants, particularly in patients with increased peripheral inflammatory biomarkers^19,20^. Elevated C-reactive protein (CRP), tumor necrosis factor alpha (TNF-α), and interleukin-6 (IL-6) are the major molecular inflammatory signature associated with depression in general population^21,22^. Among the chemical mediators, high-sensitive C-reactive protein (hsCRP) and IL-6 are mainly reported to be associated with GDM^23–25^. However, till date the association of CRP and IL-6 with depression and cardiometabolic risks in GDM has not been studied. Oxidative stress and endothelial dysfunction have also been implicated in the pathophysiology of GDM^26,27^. But the association of oxidative stress and endothelial dysfunction with stress and depression-mediated increased cardiometabolic risks in GDM has not been reported yet.

We have reported the decreased HRV as an indicator of reduced cardiovagal modulation and sympathovagal imbalance as a marker of CV risk in various disorders including diabetes^28–34^. In a recent study, we have demonstrated the link of decreased level of nitric oxide as marker of endothelial dysfunction with reduced HRV in gestational hypertension^35^. However, the extent of HRV and cardiovagal modulation and their link to depression in GDM has not been reported till date. In GDM, a cardiometabolic risk intensifies in later part of pregnancy. We have investigated the link of retrograde inflammation, oxidative stress, endothelial dysfunction and decreased cardiovagal modulation to stress and depression at 36th week of pregnancy in gestational diabetes mellitus.

## Methods

### Study design

The present descriptive-analytical study was conducted in the Department of Physiology in collaboration with the Department of Obstetrics and Gynaecology (OG), and Biochemistry, Jawaharlal Institute of Postgraduate Medical Education and Research (JIPMER), Puducherry, India from October, 2020–April, 2022 after obtaining approval from Scientific Advisory Committee and Ethics Committee of JIPMER, Puducherry. All the methods were performed in accordance with the institutional guidelines and regulations. A total of 517 pregnant women registered at admission counter of OG department of JIPMER hospital were screened. Following inclusion and exclusion criteria, 176 GDM women and 164 normal pregnant women at 36th week of gestation were recruited (as depicted in the recruitment flow-chart below).

**Figure:**
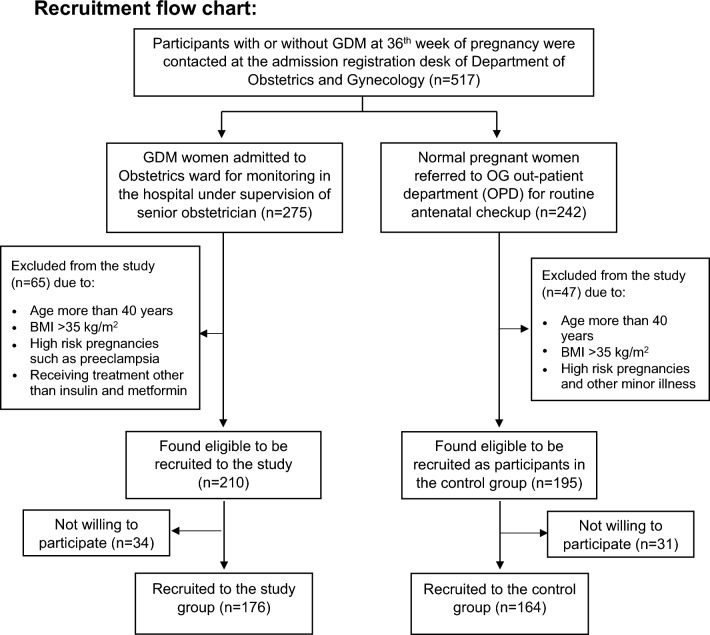

Written informed consent was obtained from all the participants prior to the initiation of the study. All the investigations were done at the bedside of the subjects in the OG Ward. GDM was diagnosed based on the International Association of Diabetes and Pregnancy Study Group (IADPSG) criteria in which 75-gm oral glucose tolerance test (OGTT) was done at 24–28 weeks in fasting and GDM was diagnosed if any one of the following cut-off is met i.e. ≥ 92 mg/dl (≥ 5.2 mmol/l) or 1-h ≥ 180 mg/dl (≥ 10 mmol/l) or 2-h ≥ 153 mg/dl (≥ 8.5 mmol/l)^36^.

### Sample size calculation

Sample size was calculated using G*Power 3.1.9.7. Based on the previous report of PSS in GDM^37^, sample size was calculated with 90% power to detect the difference between means of PSS in GDM and normal pregnant women with a significance level of (alpha) 0.01. The minimum sample size was calculated to be 106 in each group. However, since stress levels are highly subjective and HRV parameters have wider variations in pregnancy, the sample size was kept at minimum 160 in each group, in the present study.

## Grouping of subjects

### Study group (n = 176)

Subjects of study group were pregnant women diagnosed with gestational diabetes and admitted to OG ward of JIPMER hospital at 36th week of gestation.

#### Inclusion criteria

GDM women at 36th gestational weeks between the age of 18 and 40 years, and receiving insulin and/or oral anti-diabetic agents as part of anti-diabetic treatment protocol practiced in JIPMER hospital (Table 1) and followed by others^38^, in addition to the routine diabetic diet for GDM management (Table 2) and those willing to participate in the study, were included.

**Table 1:** Treatment received by pregnant women in control group and study group.

Sl. no	Control group (n = 164) (normal pregnant women)	Study group (n = 176) (women with GDM)
1	Standard antenatal medications for healthy pregnant women (i) Tab. Folate—5 mg, once daily (ii) Tab. FST—200 mg, twice daily (iii) Tab. Calcium—500 mg, once daily (iv) Tab. Vitamin C—100 mg, once daily	Standard antenatal medications for women with gestational diabetes admitted to JIPMER hospital (i) Tab. Folate—5 mg, once daily (ii) Tab. FST—200 mg, twice daily (iii) Tab. Calcium—500, mg once daily (iv) Tab. Vitamin C—100 mg, once daily
2	No medication	(i) Only insulin^a^—104 patients (ii) Both insulin^a^ and OAD^b^—47 patients (iii) Only OAD^b^—25 patients
3	No diet restriction	Diabetic diet (composition of diabetic diet was given in the table below)
4	Routine antenatal counseling	Routine antenatal counseling

^a^RI: 4U–10 U, NPH: 4U–12U, Actrapid: 6U–20U, Insulatard: 14U–18U, Mixtard: 6U–14U.

^b^Tab. Metformin 500 mg.

*GDM* gestational diabetes mellitus, *FST* ferrous sulphate tablets, *OAD* oral anti-diabetic drug.

**Table 2:** Diet given to GDM women admitted to obstetrics ward of JIPMER hospital. Three different calories of food items were given according to their individual requirements, as prescribed by the hospital nutritionist monitored on daily basis.

	Quantity	1500 calorie	1800 calorie	2000 calorie
Morning 6.30 AM				
Tea/milk without sugar	1	100 ml	150 ml	150 ml
Breakfast				
Rice/wheat/raggi (idly/dosai/upma)	1	50 g raggi	60 g raggi	60 g raggi
Sambar/chutney (tomato/onion/coriander/mint/curry leaves)	2 table spoon	15 g dal 50 g vegetable	20 g dal 50 g vegetable	25 g dal 50 g vegetable
Morning				
Buttermilk/coconut water/soup (vegetable, mushroom, corn, dal)	1 glass	200 ml	200 ml	200 ml
Lunch				
Rice/chappathi	225 g	75 g	75 g	80 g
Rasam/sambar	¾ cup	5 g dal	10 g dal	10 g dal
Chicken/fish/egg/dal	1 piece/1 katori	40 g 13 g	60 g 15 g	75 g 15 g
Greens	½ cup	100 g	100 g	100 g
Vegetable curry	2 katoris	100 g	100 g	100 g
Buttermilk	1 glass	150 ml	150 ml	150 ml
Evening				
Tea/milk (without sugar)	1 glass	100 ml	150 ml	150 ml
Vegetable sandwich/sundal	¼ cup	30 g	45 g	50 g
Dinner				
Chappathi/wheat rice	2	50 g	70 g	75 g
Dal/sambar	1 katori	10 g	10 g	10 g
Vegetable curry	2 katoris	150 g	150 g	150 g
Night				
Milk	½ glass	100 ml	100 ml	150 ml
Fruit	1	50 g	75 g	100 g
Total oil quantity		20 g	20 g	25 g
Total butter quantity				10 g
Total salt intake	Less than 6 gm			

#### Exclusion criteria

GDM women with severe anemia, polyhydramnios, multiple pregnancy, pregnancy-induced hypertension, cardiac problems, history of depression, GDM women with history of PCOS on metformin treatment and body mass index (BMI) > 35 kg/m^2^ were excluded from the study^39^.

### Control group (n = 164)

Normal pregnant women at 36th week of gestation, attending OG out-patient department (OPD) for 3rd trimester antenatal check-up, were recruited as control group participants. Subjects having BMI > 35 kg/m^2^, age more than 40 years, previous history of depression and not willing to participate were excluded from the study.

## Procedure

### Anthropometric measurements

All the subjects’ body weight (BW) in kg and height in cm were measured and body mass index (BMI) was calculated by Quetelet's index formula.

### Blood pressure-related parameters

Following 10 min of supine rest in their bed, heart rate (HR), systolic blood pressure (SBP), and diastolic blood pressure (DBP) were recorded using automated blood pressure monitor (Omron automatic blood pressure monitor HEM-8712, Omron Healthcare Company Ltd, Tokyo, Japan). Mean arterial pressure (MAP) and rate pressure product (RPP) were calculated. RPP, an index of myocardial work stress^40^ was calculated using the formula, RPP = (BHR × SBP) × 10^−2^.

### Body composition parameters

Body composition was assessed Bodystat^®^ (Model QuadScan 4000^®^, Isle of Man, United Kingdom) which works based on the bioelectrical impedance analysis (BIA). Body composition was measured following the procedure as described earlier^30^. Subjects were rested in supine position. The recording electrodes were positioned on the dorsal surface of hand proximal to metacarpal–phalangeal and on the foot proximal to metatarsal–phalangeal joints. Body composition parameters included total body fat, visceral fat, resting metabolism (RM), subcutaneous fat (whole body) and skeletal muscle (whole body), lean body mass, body fat mass index (BFMI), fat-free mass index (FFMI), and ratio of RM to body weight.

Gestational weight gain observed during pregnancy is associated with an increase in the maternal, foetal and placental tissue, changes in the amniotic and extracellular fluid, and blood volume expansion. It seems that BIA has a better prognostic potential for gestational and post-partum outcomes than body mass index^41^. It has been observed that the BIA method can be successfully used to study the effect of excessive gestational weight gain in pregnancy on the development of obstetric complications, including gestational diabetes mellitus^42^^.^

### HRV recording

Following 10 min of rest in supine position, short-term HRV was recorded based on the recommendation of Task Force on HRV^43^ and as described earlier^35^. ECG electrodes were connected and Lead II ECG was acquired for 15 min at a rate of 20,000 samples/sec using BIOPAC MP 46 data acquisition system (BIOPAC Inc., USA). The data were transferred from BIOPAC to a laptop with Biopac Student Lab (BSL) software. With due care, ectopics and artifacts were meticulously removed from the recorded ECG. By using the R wave detector in the BSL software, the extraction of RR tachogram was done from the edited 900 s ECG. HRV analysis was done by using software (Kubios HRV standard, version 3.5.0) that analyzes the frequency spectral components and time-domain components using Fast Fourier transformation in the RR trend. The frequency-domain indices of HRV recorded were total power (TP), component of low frequency expressed as normalized unit (LFnu), component of high frequency expressed as normalized unit (HFnu), and LF–HF ratio. The time-domain indices of HRV included square root of the mean of the sum of the squares of differences between adjacent RR interval (RMSSD), mean and standard deviation of RR intervals (SDNN), adjacent RR interval differing more than 50 ms (NN50), and NN50 counts divided by all RR intervals (pNN50).

### Perceived stress scale

Perceived Stress Scale (PSS) was used to determine the stress level of the subjects^17,44^. The questionnaire consists of 10 questions with a score of 0–4 each. The score ranges from 0 to 40. A score of 0–13 is low stress, 14–26 is moderate stress and 27–40 is considered as high stress.

### Edinburgh postnatal depression scale

Edinburgh Postnatal Depression Scale (EPDS) was used to assess the participant mood in the past 1 week^17,45^. It entails ten questions with a score of 0–3 each question. The score of EPDS ranges from 0 to 30. The higher the scores, risk of depression is high and a score of 10 or greater indicates possible depression.

### Flanagan’s quality of life

In the present study, Flanagan Quality of Life (QoL) scale was used to assess the quality of life of the participants^46^. The questionnaire comprises of 16 items covering five aspects including physical and Material well-being; relations with other people; social, community and civic activities; personal development and fulfilment; and recreation. The score ranges from 16 to 112.

Participants were not asked to fill the questionnaires on their own. However, to avoid biased information, an independent researcher was assigned for the task. Time taken to fill the above-mentioned three set of questionnaires was about 15 to 20 min.

### Estimation of biochemical parameters

Five ml of fasting blood samples were collected and serum was separated and stored at − 80 °C for further analysis. Fasting blood glucose (FBG) was noted from the patients’ case records. Insulin was measured using enzyme linked immunosorbent assay (ELISA) (Calbiotech, USA). For determination of insulin resistance, homeostatic model assessment (HOMA) of insulin resistance was calculated [HOMA-IR = FBG (mg/dL) × Insulin (μIU/L)/405]. HbA1c was determined from whole blood using commercial kits for turbidimetric immunoassay (Quantia, Tulip diagnostics). Malondialdehyde (MDA), the oxidative stress marker was measured by colorimetric assay kit (Elabscience, USA) and inflammatory markers such as interleukin-6 (IL-6) (Diaclone, France) and high sensitive C-reactive protein (hsCRP) (Calbiotech, USA) were measured by ELISA kits according to the manufacturer instructions. Nitric oxide derivatives (nitrate and nitrite) were estimated by colorimetric method using microplate reader (Elabscience, USA).

### Statistical analysis

Statistical Package for the Social Sciences (SPSS) version 13 (SPSS Software Inc., Chicago, IL, USA) was used for statistical analysis. Normality of data was tested by Kolmogorov–Smirnov test and accordingly data were expressed as mean ± SD and median (interquartile range) for parametric and non-parametric data respectively. For comparison of data between control and study groups, the level of significance was tested by unpaired *t* test for parametric data, Mann–Whitney U test for non-parametric data. The association of PSS and EPDS with various parameters was assessed by Spearman correlation analysis. The independent contribution of various parameters to PSS and EPDS was assessed by multiple regression analysis. The *p* value less than 0.05 were considered statistically significant.

### Ethics approval and consent to participate

Approval was obtained from Institute Ethics Committee (Human studies) of JIPMER (Reference number: JIP/IEC/2020/09). All the methods were performed in accordance with the institutional guidelines and regulations. Individual written informed consent was obtained from the participants before recruiting into the study.

## Results

There was no significant difference in age between the subjects of control group and study group. There was a significant increase (*p* < 0.001) in the body weight and BMI in the study group compared to control group (Table 3).

**Table 3:** Comparison of anthropometric, body composition and blood pressure-related parameters in normal pregnant women (control group) and women with GDM (study group) at 36th week of gestation.

Parameters	Control group (n = 164)	Study group (n = 176)	*p* value
Age (years)	27.35 (24.00–30.00)	27.50 (25.00–30.00)	0.764
Body weight (kg)	65.41 ± 8.78	71.04 ± 12.37	< 0.001
BMI (kg/m^2^)	25.80 (22.70–28.80)	28.29 (26.22–31.37)	< 0.001
Body composition parameters			
Total body fat (%)	27.12 ± 4.38	33.10 ± 5.55	< 0.001
Visceral fat (%)	5.86 ± 2.10	7.91 ± 2.71	< 0.001
Body lean (%)	72.85 ± 7.15	66.17 ± 6.80	< 0.001
RM (kcal/day)	1245.70 ± 136.90	1424.03 ± 182.60	< 0.001
BFMI (kg/m^2^)	7.38 ± 0.88	9.67 ± 1.08	< 0.001
FFMI (kg/m^2^)	19.75 ± 1.37	20.26 ± 1.42	0.001
Subcutaneous fat (%)	27.70 (22.50–33.20)	31.50 (26.40–35.67)	< 0.001
Skeletal muscle (%)	26.77 ± 2.95	24.49 ± 2.98	< 0.001
RM/BW (kcal/kg)	19.03 ± 1.85	20.49 ± 2.56	< 0.001
RM/BF (kcal/kg)	71.02 ± 7.45	60.56 ± 6.10	< 0.001
Blood pressure related parameters			
HR (per min)	80.12 ± 7.30	90.15 ± 10.07	< 0.001
SBP (mmHg)	111.00 (103.00–119.00)	120.00 (110.00–125.00)	< 0.001
DBP (mmHg)	71.00 (65.00–79.00)	78.00 (70.00–83.00)	< 0.001
MAP (mmHg)	84.95 (77.50–92.80)	91.67 (83.75–96.33)	< 0.001
RPP (mmHg)	90.85 (78.85–105.20)	106.09 (98.28–116.61)	< 0.001

The data presented are mean ± SD and median (IQR). Comparison was done by unpaired *t* test for parametric data and Mann Whitney U test for non-parametric data. *P* value < 0.05 was considered statistically significant.

*BMI* body mass index, *RM* resting metabolism, *BFMI* body fat mass index, *FFMI* fat-free mass index, *BW* body weight, *BF* body fat, *HR* heart rate, *SBP* systolic blood pressure, *DBP* diastolic blood pressure, *MAP* mean arterial pressure, *RPP* rate pressure product.

Body composition parameters such as total body fat, visceral fat, RM, BFMI, FFMI (*p* = 0.001), subcutaneous fat and RM/BW were found to be significantly increased (*p* < 0.001) and body lean, skeletal muscle and RM/BF were found to be significantly decreased (*p* < 0.001) in study group compared to control group (Table 3). HR, SBP, DBP, MAP, and RPP of study subjects were significantly increased (*p* < 0.001) in study group compared to the control group subjects (Table 3).

Among the frequency domain indices of HRV, TP and HFnu were significantly reduced (*p* < 0.001), and LFnu and LF–HF ratio were significantly increased (*p* < 0.001) in study group compared to control group subjects. Time domain indices such as SDNN, RMSSD, NN50 and pNN50 were significantly reduced (*p* < 0.001) in subjects of study group compared to control group (Table 4).

**Table 4:** Comparison of frequency domain and time domain indices of heart rate variability, perceived stress scale, quality of life and Indian diabetic risk score in normal pregnant women (control group) and women with GDM (study group) at 36th week of gestation.

Parameters	Control group (n = 164)	Study group (n = 176)	*p* value
HRV frequency domain indices			
TP (ms^2^)	950.74 (610.20–1310.20)	506.50 (393.00–597.00)	< 0.001
LFnu	50.50 (38.70–62.36)	62.04 (51.85–65.56)	< 0.001
HFnu	46.90 (40.25–61.86)	37.74 (33.65–47.93)	< 0.001
LF–HF ratio	1.10 (0.72–1.40)	1.62 (1.08–1.93)	< 0.001
HRV time domain indices			
SDNN (ms)	40.10 (27.35–52.10)	21.20 (17.40–29.70)	< 0.001
RMSSD (ms)	45.32 (31.20–58.80)	23.70 (18.40–30.30)	< 0.001
NN50	33.15 (25.20–43.62)	28.00 (22.00–32.00)	< 0.001
pNN50 (%)	10.27 (7.10–13.20)	6.05 (4.59–6.88)	< 0.001
Stress and risk scores			
PSS score	14.30 (11.50–17.80)	24.00 (21.00–27.75)	< 0.001
EPDS score	7.60 ± 1.80	10.57 ± 2.21	< 0.001
Flanagan QoL score	105.00 (93.00–113.50)	90.00 (82.25–95.00)	< 0.001
IDRS	40.00 (35.20–45.80)	60.00 (50.00–70.00)	< 0.001

The data presented are mean ± SD and median (IQR). Comparison was done by unpaired *t* test for parametric data and Mann Whitney U test for non-parametric data. *P* value < 0.05 was considered statistically significant.

*TP* total power of HRV, *LFnu* low-frequency power normalized, *HFnu* high-frequency power normalized, *LF–HF* *ratio* ratio of LF to HF, *SDNN* standard deviation of normal to normal (NN) interval, *RMSSD* square root of the mean of the sum of the squares of the differences between adjacent NN intervals, *NN50* number of interval differences of successive NN intervals greater than 50, *pNN50* NN50 counts divided by all RR intervals, *PSS* perceived stress scale, *EPDS* Edinburgh postnatal depression scale,* QoL* quality of life, *IDRS* Indian diabetic risk score.

PSS score, EPDS and IDRS were significantly increased in study group (*p* < 0.001) when compared to compared group, whereas QoL score was significantly reduced (*p* < 0.001) in study group than control group subjects (Table 4).

Glucose-related parameters such as FBG, insulin, HOMA-IR and HbA1C were significantly increased (*p* < 0.001) in study group compared to control group. Inflammatory markers such as IL-6 and hsCRP, and the oxidative stress marker, MDA were significantly increased (*p* < 0.001), while NO was significantly decreased (*p* < 0.001) in the subjects of study group compared to control group (Table 5).

**Table 5:** Comparison of insulin resistance markers, oxidative stress marker and inflammatory markers in normal pregnant women (control group) and women with GDM (study group) at 36th week of gestation.

Parameters	Control group (n = 164)	Study group (n = 176)	*p* value
Glucose-related parameters			
FBG (mg/dL)	86.17 ± 12.45	116.86 ± 16.62	< 0.001
Insulin (uIU/ml)	10.60 (5.10–16.80)	23.85 (16.38–31.29)	< 0.001
HOMA-IR	3.20 (1.10–5.20)	6.33 (4.16–9.03)	< 0.001
HbA1C (gm%)	5.18 ± 0.86	6.81 ± 1.19	< 0.001
Oxidative stress marker			
MDA (μmol/L)	15.10 (11.20–19.80)	22.95 (17.62–25.60)	< 0.001
Inflammatory markers			
IL-6 (pg/mL)	12.45 ± 3.05	20.12 ± 5.34	< 0.001
hsCRP (μmol/L)	3.10 (2.15–5.10)	7.28 (4.71–9.65)	< 0.001
Endothelial dysfunction marker			
NO (µM/L)	30.80 ± 3.80	25.43 ± 2.83	< 0.001

The data presented are mean ± SD and median (IQR). Comparison was done by unpaired *t* test for parametric data and Mann Whitney U test for non-parametric data. *P* value < 0.05 was considered statistically significant.

*FBG* fasting blood glucose, *HOMA-IR* homeostatic model assessment-insulin resistance, *HbA1C* glycated hemoglobin, *MDA* malondialdehyde, *IL-6* interleukin-6, *hsCRP* high sensitive c-reactive protein, *NO* nitric oxide.

**Figure 1:**
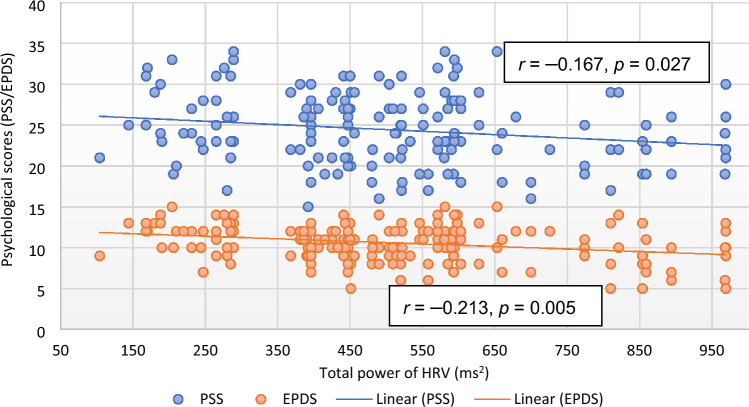
Correlation of total power of heart rate variability with psychological scores. *EPDS* Edinburgh postnatal depression scale, *HRV* heart rate variability, *PSS* perceived stress scale.

**Figure 2:**
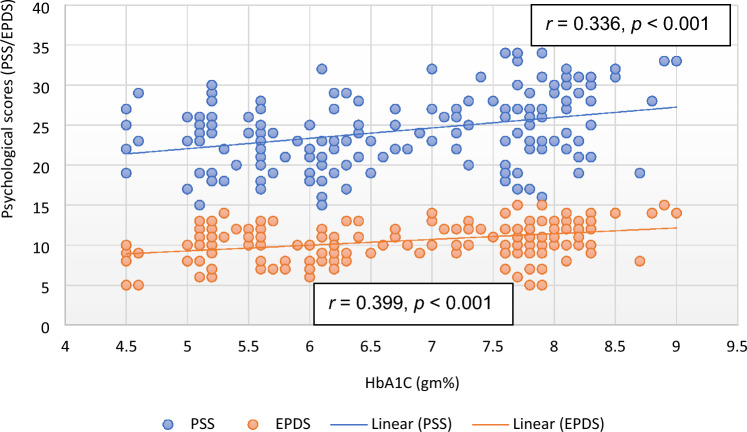
Correlation of glycated hemoglobin with psychological scores. EPDS Edinburgh postnatal depression scale, HbA1C glycated hemoglobin, *PSS* perceived stress scale.

**Figure 3:**
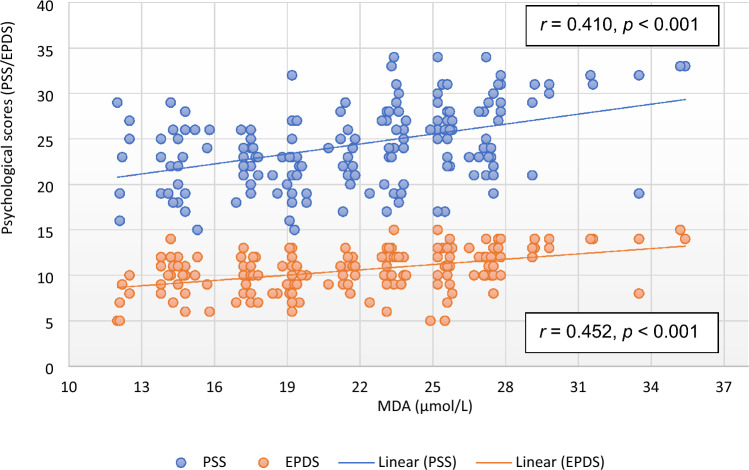
Correlation of malondialdehyde with psychological scores. *EPDS* Edinburgh postnatal depression scale, *MDA* malondialdehyde, *PSS* perceived stress scale.

**Figure 4:**
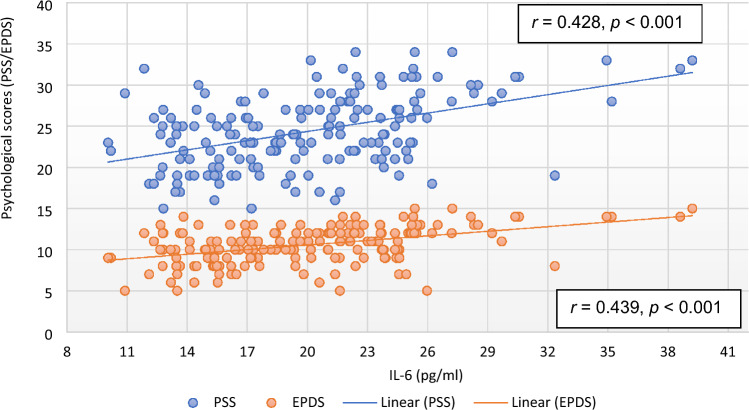
Correlation of interleukin-6 with psychological scores. *EPDS* Edinburgh postnatal depression scale, *IL-6* interleukin-6, *PSS* perceived stress scale.

Except EPDS and QoL, there was no significant correlation of PSS with any of the parameter in the subjects of control group. However, the correlation of RPP (*r* = 0.193, *p* = 0.010), RM (*r* = 0.151, *p* = 0.045), HRV parameters [TP (*r* =  − 0.167, *p* = 0.027; Fig. 1), LFnu (*r* = 0.235, *p* = 0.002), LF–HF ratio (*r* = 0.184, *p* = 0.014)], HbA1C (*r* = 0.336, *p* < 0.001; Fig. 2), oxidative stress marker [MDA (*r* = 0.410, *p* < 0.001; Fig. 3)], inflammatory marker, IL-6 (*r* = 0.428, *p* < 0.001; Fig. 4), endothelial dysfunction marker, NO (*r* =  − 0.381, *p* < 0.001) and depression and risk scores such as EPDS (*r* = 0.582, *p* < 0.001), QoL (*r* =  − 0.299, *p* < 0.001) and IDRS (*r* = 0.195, *p* = 0.009) with PSS were significant in the study group (Table 6).

**Table 6:** Spearman correlation of PSS with various parameters in control group (n = 164) and study group (n = 176) at 36th week of gestation.

Parameters	Control group at 36th week		Study group at 36th week	
	*r* value	*p* value	*r* value	*p* value
BMI	–0.045	0.398	0.013	0.867
HR	0.109	0.185	0.120	0.114
SBP	–0.145	0.095	0.090	0.235
DBP	–0.102	0.197	0.077	0.310
RPP	–0.152	0.085	0.193	0.010
RM	0.115	0.157	0.151	0.045
TP	0.108	0.186	–0.167	0.027
LFnu	0.099	0.192	0.235	0.002
HFnu	0.053	0.286	–0.140	0.064
LF–HF ratio	–0.098	0.217	0.184	0.014
HbA1C	0.110	0.175	0.336	< 0.001
MDA	–0.103	0.195	0.410	< 0.001
hsCRP	–0.083	0.213	0.109	0.149
IL-6	–0.110	0.183	0.428	< 0.001
NO	0.154	0.088	–0.381	< 0.001
EPDS	0.198	0.024	0.582	< 0.001
QoL	0.162	0.048	–0.299	< 0.001
IRDS	–0.095	0.220	0.195	0.009

The *p* value < 0.05 was considered significant; *r:* correlation coefficient.

*PSS* perceived stress scale, *BMI* body mass index, *HR* heart rate, *SBP* systolic blood pressure, *DBP* diastolic blood pressure, *RPP* rate pressure product, RM resting metabolism, *TP* total power of HRV, *LFnu* low-frequency power normalized, *HFnu* high-frequency power normalized, *LF–HF* ratio of LF to HF, *HbA1C* glycated hemoglobin, *MDA* malondialdehyde, *hsCRP* high sensitive c-reactive protein, *IL-6* interleukin-6, *NO* nitric oxide, *EPDS* Edinburgh postnatal depression scale, *QoL* quality of life, *IRDS* Indian diabetic risk score.

Except PSS, none of the parameters were significantly correlated with EPDS in the subjects of control group. In study group subjects, BMI, HR, SBP and DBP were not significantly correlated with EPDS scores, however, RPP (*r* = 0.168, *p* = 0.026) and RM (*r* = 0.153, *p* = 0.043) showed significant positive correlation to EPDS scores (Table 7).

**Table 7:** Spearman correlation of EPDS with various parameters in control group (n = 164) and study group (n = 176) at 36th week of gestation.

Parameters	Control group at 36th week		Study group at 36th week	
	*r* value	*p* value	*r* value	*p* value
BMI	–0.110	0.184	0.119	0.115
HR	0.093	0.202	0.109	0.185
SBP	–0.106	0.162	0.145	0.095
DBP	–0.080	0.210	0.147	0.051
RPP	–0.160	0.072	0.168	0.026
RM	0.102	0.195	0.153	0.043
TP	0.115	0.172	–0.213	0.005
LFnu	0.090	0.192	0.287	< 0.001
HFnu	0.050	0.302	–0.201	0.007
LF–HF ratio	–0.090	0.232	0.236	0.002
HbA1C	0.112	0.181	0.399	< 0.001
MDA	–0.150	0.086	0.452	< 0.001
hsCRP	–0.045	0.318	0.186	0.013
IL-6	–0.142	0.112	0.439	< 0.001
NO	0.158	0.078	–0.419	< 0.001
PSS	0.180	0.047	0.582	< 0.001
QoL	0.048	0.310	–0.163	0.035
IRDS	–0.175	0.052	0.178	0.018

The *p* value < 0.05 was considered significant; *r:* correlation coefficient.

*EPDS* Edinburgh postnatal depression scale, *HR* heart rate, *SBP* systolic blood pressure, *DBP* diastolic blood pressure, *RPP* rate pressure product, *RM* resting metabolism, *TP* total power of HRV, *LFnu* low-frequency power normalized, *HFnu* high-frequency power normalized, *LF–HF* ratio of LF to HF, *HbA1C* glycated hemoglobin, *MDA* malondialdehyde, *hsCRP* high sensitive c-reactive protein, *IL-6* interleukin-6, *NO* nitric oxide, *PSS* perceived stress scale, *QoL* quality of life, *IRDS* Indian diabetic risk score.

IL-6 (*r* = 0.439, *p* < 0.001; Fig. 4), MDA (*r* = 0.452, *p* < 0.001; Fig. 3), HbA1C (*r* = 0.399, *p* < 0.001; Fig. 2), hsCRP (*r* = 0.186, *p* < 0.013), were positively correlated and NO (*r* =  − 0.419, *p* < 0.001) was negatively correlated with EPDS scores in subjects of study group. Among HRV parameters, TP (*r* =  − 0.213, *p* = 0.005; Fig. 1) and HFnu (*r* =  − 0.201, *p* = 0.007) were negatively correlated and LFnu (*r* = 0.287, *p* < 0.001) and LF–HF ratio (*r* = 0.236, *p* < 0.002) were positively correlated with EPDS scores. PSS (*r* = 0.582, *p* < 0.001) and IDRS (*r* = 0.178, *p* = 0.018) were positively correlated and QoL (*r* =  − 0.163, *p* = 0.035) was negatively correlated with EPDS (Table 7).

Multiple regression analysis revealed significant individual contribution of RPP (β = 0.154, *p* = 0.021), TP (β =  − 0.182, *p* = 0.005), HbA1C (β = 0.386, *p* = 0.016), MDA (β = 0.345, *p* = 0.004) and IL-6 (β = 0.550, *p* < 0.001) to PSS in study group. Though RM and NO were correlated with PSS, its independent contribution to PSS was not significant (Table 8). TP (β =  − 0.286, *p* < 0.001), HbA1C (β = 0.237, *p* = 0.004), MDA (β = 0.337, *p* < 0.001) and IL-6 (β = 0.393, *p* < 0.001) significantly contributed to EPDS in study group (Table 9).

**Table 8:** Multiple regression analysis of PSS (as dependent variable) with various parameters (as independent variables) in study group at 36th week of gestation, adjusted for plasma insulin.

Independent variables	Standardized regression coefficient β	95% C.I.		*p* value
		Lower limit	Upper limit	
RPP	0.154	0.007	0.085	0.021
TP	–0.182	–0.007	–0.001	0.005
RM	0.117	0.000	0.006	0.077
HbA1C	0.386	–2.560	–0.270	0.016
MDA	0.345	0.096	0.492	0.004
IL-6	0.550	0.221	0.681	< 0.001
NO	–0.035	–0.415	0.524	0.819

R: 0.578; R^2^: 0.334; adjusted R^2^: 0.307.

*C.I.* confidence interval, *PSS* perceived stress scale, *RPP* rate pressure product, *TP* total power of HRV, *RM* resting metabolism, *HbA1C* glycated hemoglobin, *MDA* malondialdehyde, *IL-6* interleukin-6, *NO* nitric oxide.

**Table 9:** Multiple regression analysis of EPDS (as dependent variable) with various parameters (as independent variables) in study group at 36th week of gestation, adjusted for plasma insulin.

Independent variables	Standardized regression coefficient β	95% C.I.		*p* value
		Lower limit	Upper limit	
RPP	0.105	–0.004	0.035	0.112
TP	–0.286	–0.005	–0.002	< 0.001
RM	0.072	–0.001	–0.002	0.272
HbA1C	0.237	–1.009	0.133	0.004
MDA	0.337	0.046	0.243	< 0.001
IL-6	0.393	0.048	0.277	< 0.001
NO	–0.010	–0.242	0.227	0.948

R: 0.591; R^2^: 0.349; adjusted R^2^: 0.322.

*C.I.* confidence interval, *EPDS* Edinburgh postnatal depression scale, *RPP* rate pressure product, *TP* total power of HRV, *RM* resting metabolism, *HbA1C* glycated hemoglobin, *MDA* malondialdehyde, *IL-6* interleukin-6, *NO* nitric oxide.

## Discussion

The participants of study group of the present study were GDM patients admitted to obstetrics ward of JIPMER hospital, which is a tertiary healthcare center of central government of India. Usually, the GDM patients admitted to this tertiary care hospital few weeks before delivery are those who have poor glycemic control inspite of treatment or those who are at risk of developing complications during delivery; otherwise they come for delivery just a couple of days before the expected date of delivery or after developing the labour pain. Thus, the GDM women in the study group had relatively less glycemic control in their 3rd trimester of pregnancy inspite of receiving antidiabetic treatment since the diagnosis of GDM. In the present study, the stress and depression scores were significantly increased in study group subjects compared to control group subjects depicting that the perceived psychological stress and depression are more in 36th week of gestation in pregnant women diagnosed with GDM. Earlier report^37^ suggests that pregnant women with GDM experience higher levels of anxiety and stress compared to non-pregnant women and healthy pregnant women. Diagnosis of GDM in pregnant women increases their anxiety, resulting in 2–4 times susceptibility to have antenatal or postnatal depression than women without GDM during pregnancy^47^. Pace et al^48^, reported that when pregnant women with GDM become apprehensive of uncontrolled diabetes resulting in adverse maternal and neonatal outcomes, they are more likely to experience antenatal depression, stress and anxiety^49^. In these women, the causes of anxiety and depression could be the inability to manage diabetes, serious concerns about the disease, need for dietary restrictions, insulin treatment and the demand for self-assessment of blood glucose^50^. Also, GDM patients are afraid of treatment and are concerned about the adverse effects of medication on the growth and development of the fetus, resulting in anxiety^51^. Additionally, women who received insulin treatment experienced significantly higher levels of stress compared to women who only received dietary treatment alone^51^. In the present study, 85.79% GDM patients (151/176) had received insulin therapy in addition to dietary management (Table 1), which could have been a factor for stress and depression in these patients.

In our current study, along with increased stress and depression there was decreased quality of life (QoL) in the study group compared to the control group subjects (Table 4). QoL is the most important indicator for assessing the status of health care in chronic diseases such as diabetes and it has been reported that increased stress is linked to poor QoL in diabetes^52^. Though decreased QoL has been reported in GDM^53,54^, the relationship of stress to QoL has not been studied in GDM yet. In our study, PSS (Table 6) and EPDS (Table 7) were highly correlated with decreased QoL in GDM women at 36th week of gestation. It has been observed earlier that depression in diabetes is an important comorbidity that requires careful management because of its severe impact on QoL^55^. As such, decreased QoL increases the cardiovascular risk^56^. Therefore, the health care professionals need to consider these findings when treating patients with gestational diabetes. Further, in the present study, IDRS was significantly high in study group compared to control group and IDRS was significantly correlated with PSS (Table 6) and EPDS (Table 7) indicating that GDM women are at the higher risk of developing metabolic syndrome, which is defined as the cluster of conditions consisting of hyperinsulinemia (the upper fourth of the fasting insulin level among nondiabetic subjects) or hyperglycemia (fasting glucose ≥ 110 mg/dl) in addition to at least two of the following: waist girth ≥ 94 cm, dyslipidemia (triglycerides ≥ 150 mg/dl or HDL cholesterol < 40 mg/dl), or BP ≥ 140/90 mmHg or taking BP medication^57^. Though IDRS is generally used for screening of diabetes in the community^58^, the higher IDRS is reported to be associated with higher risk of metabolic syndrome and CVD even among people without prediabetes or diabetes^59^. Thus, decreased QoL and increased IDRS as observed in the study group subjects in the present study could increase the risk of developing CVD in women having GDM.

Though stress and depression have been highly documented in GDM, the pathophysiologic mechanisms of development of these mental illnesses in women with GDM have not been well studied till date. In the present study, the markers of inflammation were significantly increased in study group population (Table 5). Reports of animal studies and clinical trials indicate that among pro-inflammatory cytokines, IL-6 plays a unique role in the pathophysiology and somatic consequences of depression, and also mediate the effects of treatment of depressive disorder^60,61^. The IL-6 was highly correlated with PSS (*r* = 0.428, *p* < 0.001; Table 6) and EPDS (*r* = 0.439, *p* < 0.001; Table 7) (Fig. 4) and among all the parameters, IL-6 had highest independent contribution to PSS (β = 0.550, *p* < 0.001, Table 8) and EPDS (β = 0.393, *p* < 0.001, Table 9). Thus, findings of our study indicate that inflammation is associated with GDM, and IL-6 could be the potential contributor to the development of stress and depression in GDM. It has been stated earlier that elevated IL-6 activity may lead to depression through stimulation of hypothalamic–pituitary–adrenal axis or by influencing the metabolism of neurotransmitters^60,62^. Other suggested mechanisms are that IL-6 may increase the activity of indoleamine-2,3-dioxygenase (IDO), which catalyses tryptophan that in turn activate kynurenine pathway and reduce availability of serotonin in the brain^63,64^. As a result, quinolinic acid, 3-hydroxy kynurenine, and the neurotoxic *N*-methyl-d-aspartate glutamate agonist are produced, which causes oxidative stress and contributes to the neurodegeneration and depression. IL-6 is also reported to be involved of in neuro-inflammation and brain-derived neurotropic factor (BDNF)-mediated depression in various mental disorders^65^. Therefore, future studies should focus on the association of IL-6 with BDNF and brain activity of serotonin and IDO to understand the role of neuro-inflammation in the causation of depression in GDM.

Malondialdehyde, the marker of oxidative stress was significantly increased in study group compared to control group. Malondialdehyde was significantly correlated with stress and depression (Fig. 3) and had independent contribution to both PSS (Table 8) and EPDS (Table 9). These findings indicate the possible role of oxidative stress in causation of stress and depression in GDM. Though oxidative stress has been implicated in poor fetomaternal outcomes in GDM^66^, its role in pathophysiology of stress and depression in GDM has not been reported yet. Nitric oxide, the marker of endothelial dysfunction was significantly increased in study group and was substantially correlated with both stress and depression (Tables 6 and 7). However, nitric oxide had no independent association with PSS (Table 8) and EPDS (Table 9). Though nitric oxide is known to be involved in the pathophysiology of cardiometabolic risks in GDM both in antepartum and postpartum periods^67^, from the finding of the present study it appears that endothelial dysfunction is unlikely to be linked to stress and depression in GDM.

FBG and HbA1C were significantly high in study group compared to control group, indicating that glycemic control was not effective inspite of antidiabetic treatment given to all GDM patients (Table 1). Glycated hemoglobin was strongly correlated with both stress and depression (Tables 6 and 7; Fig. 2) and had strong independent association with both the parameters. The level of glycated hemoglobin generally reflects the glycemic status in last three months period of a person and a higher glycated hemoglobin represents the poor diabetic control and increased cardiovascular risk^68^. The higher blood glucose and HbA1C levels has been reported to be associated with higher stress in pregnant women^69^. Thus, it appears that disease severity (level of glycemic status) could be an indicator of stress and depression in GDM and higher glycated hemoglobin level could herald the onset of depression and cardiovascular disease in GDM. As 151 out of 176 GDM patients received insulin therapy (Table 1), HOMA-IR was not considered for correlation and regression analysis. Also, plasma insulin was adjusted in the multiple regression models (Tables 8 and 9).

Spectral analysis of HRV has been used for assessment of autonomic functions and dysfunctions in health and diseases^28–31^ and decreased HRV is a known marker of CVD risk^33,34,70^. In the present study, TP of HRV was considerably decreased in study group and control group (Table 4) and TP had significant correlation (Tables 6 and 7; Fig. 1) and independent association with stress and depression scores (Tables 8 and 9). TP of HRV reflects the overall magnitude of variability of heart rate and the vagal drive of cardiac modulation^43,71^. Decreased TP is an established marker of CVD risk^71^. Decrease in time domain indices of HRV also reflects decreased cardiac vagal modulation^71^. The time-domain indices were significantly decreased in study group subjects (Table 4). Thus, findings of the present study reveal the decreased cardiovagal modulation at 36th week of gestation in women with GDM, which is linked to increased stress and depression in women those who have been receiving antidiabetic treatment for GDM. Increased LF–HF ratio, the marker of sympathovagal imbalance was prominently higher in study group compared to control group, indicating a severe state of autonomic dysregulation in women with GDM. To best of our knowledge, the present study is the first of its kind linking the reduction in HRV and cardiometabolic risks with depression in GDM.

In the present study, RPP was significantly more in study group subjects, and increased RPP was significantly correlated (Tables 6 and 7) and independently associated (Tables 8 and 9) with PSS and EPDS. Increase in RPP physiologically indicates myocardial work stress^40^. Increase in RPP and heart rate has reported to be associated with CVD risks and sudden cardiac death, especially in individuals having high BP^72^. In the present study, HR and SBP were significantly more in study group subjects compared to control group subjects. Thus, findings of the present study indicate the presence of increased myocardial work stress in GDM and its possible link to stress and depression in GDM. Though BMI, body fat%, visceral fat%, BFMI, FFMI and resting metabolism were significantly high in the study group compared to control group (Table 3), the body composition and metabolism had no influence on stress and depression (Tables 8 and 9).

All the patients were followed upto delivery and their two important fetomaternal outcomes were noted. In control group, 82.92% (136/164) had normal delivery and 17.08% (28/164) had caesarean delivery, whereas in study group, 55.11% (97/176) had normal delivery and 44.89% (79/176) had caesarean delivery. The birth weight was normal (with reference to Indian standard birth weight of 2.7 kg to 3.1 kg in females, and 2.8 kg to 3.2 kg in males) in 92.68% (152/164) neonates in control group (birth weight 2.94 ± 0.24 kg) and there was no incidence of macrosomia. In study group, 64.77% (114/176) neonates had normal birth weight (2.97 ± 0.27 kg) and 48 neonates had macrosomia, i.e., more than 3.5 kg as per Indian birth weight reference^73^. Thus, it appears that fetomaternal outcomes were poor in women with GDM.

Persistent increase in IL-6 has been reported in stress reactions and in patients with depression. In the present study, the increased IL-6 were found to be strongly associated with stress and depression in GDM women receiving antidiabetic treatment. All these evidence suggest for future studies to address if restoration of IL-6 level could be the key to treatment of depression associated with inflammation in GDM. In developing countries like India, the glycemic control and fetomaternal outcomes are poor in GDM due to various socio-economic factors that include poor compliance to treatment^54,73^. Therefore, studies should be designed to assess if non-pharmacological interventions like yoga instituted early in the pregnancy can improve cardiovagal modulation and prevent the development of stress and depression in GDM.

### Limitations

The major limitation of the study is that we did not recruit the subjects in 24th to 28th week of gestation to assess the cardiometabolic risks in GDM women when they were diagnosed with the disease. Also, we have not assessed the details of fetomaternal outcomes. However, the present study reports the cardiometabolic risks in GDM women at 36th week of pregnancy, and their potential contributions to stress and depression in GDM.

## Data Availability

The datasets used and/or analysed during the current study are available from the corresponding author on reasonable request.

## Abbreviations

GDM: Gestational diabetes mellitus
CMR: Cardiometabolic risks
BP: Blood pressure
HRV: Heart rate variability
HbA1C: Glycated hemoglobin
PSS: Perceived stress score
QoL: Quality of life
IDRS: Indian diabetic risk score
EPDS: Edinburg postnatal depression score
MDA: Malondialdehyde
TP: Total power
CV: Cardiovascular
TNF-α: Tumor necrosis factor alpha
IL-6: Interleukin-6
hsCRP: High-sensitive C-reactive protein
IADPSG: International association of diabetes and pregnancy study group
OGTT: Oral glucose tolerance test
BW: Body weight
BMI: Body mass index
HR: Heart rate
SBP: Systolic blood pressure
DBP: Diastolic blood pressure
MAP: Mean arterial pressure
RPP: Rate pressure product
BIA: Bioelectrical impedance analysis
RM: Resting metabolism
BFMI: Body fat mass index
FFMI: Fat free mass index
LFnu: Low frequency power expressed in normalized units
HFnu: High frequency power expressed in normalized units
LF–HF ratio: Ratio of low-frequency to high-frequency power
SDNN: Mean and standard deviation of RR intervals
RMSSD: Square root of the mean of the sum of the squares of differences between adjacent RR interval
NN50: Adjacent RR interval differing more than 50 ms
pNN50: NN50 counts divided by all RR intervals
HOMA: Homeostatic model assessment

## References

[1] American Diabetes Association (2003). Gestational diabetes mellitus. Diab. Care..

[2] International Diabetes Federation. *IDF Diabetes Atlas*. 7th ed. http://www.idf.org/diabetesatlas.

[3] Chudasama RK, Kadri AM, Ratnu A, Jain M, Kamariya CP (2019). Magnitude of gestational diabetes mellitus, its influencing factors and diagnostic accuracy of capillary blood testing for its detection at a tertiary care centre, Rajkot, Gujarat. Indian J. Commun. Med..

[4] Kulshrestha V, Agarwal N (2016). Maternal complications in pregnancy with diabetes. J. Pak. Med. Assoc..

[5] Chong S, Yu-Mei W, Chen W, Hui-Xia Y (2019). Updates in long-term maternal and fetal adverse effects of gestational diabetes mellitus. Matern. Fetal Med..

[6] Wicklow, B., Retnakaran R. Gestational diabetes mellitus and its implications across the life span. *Diabetes Metab J*. (2023). 10.4093/dmj.2022.0348 PMC10244196 36750271

[7] Biaggi A, Conroy S, Pawlby S, Pariante CM (2016). Identifying the women at risk of antenatal anxiety and depression: A systematic review. J. Affect. Disord..

[8] Ross GP (2016). Relationship between depression and diabetes in pregnancy: A systematic review. World J. Diab..

[9] Lydon K (2012). Psychological stress associated with diabetes during pregnancy: A pilot study. Ir. Med. J..

[10] Beka Q (2018). Development of perinatal mental illness in women with gestational diabetes mellitus: A population-based cohort study. Can. J. Diab..

[11] Riggin L (2020). Association between gestational diabetes and mental illness. Can. J. Diab..

[12] Yang HO, Chen BO, Abdulrahman AM, Li L, Wu N (2021). Associations between gestational diabetes and anxiety or depression: A systematic review. J. Diabetes Res..

[13] Byrn MA, Penckofer S (2013). Antenatal depression and gestational diabetes: A review of maternal and fetal outcomes. Nurs. Womens Health..

[14] Cosson E (2015). Psychosocial deprivation in women with gestational diabetes mellitus is associated with poor fetomaternal prognoses: An observational study. BMJ Open.

[15] Ruohomäki A (2018). The association between gestational diabetes mellitus and postpartum depressive symptomatology: A prospective cohort study. J. Affect. Disord..

[16] Green JB (2021). Cardiovascular consequences of gestational diabetes. Circulation.

[17] Shah Z, Pal P, Pal GK, Papa D, Bharadwaj B (2020). Assessment of the association of heart rate variability and baroreflex sensitivity with depressive symptoms and stress experienced by women in pregnancy. J. Affect. Disord..

[18] Renugasundari M (2021). Assessment of a short-course yoga practice on cardiometabolic risks, fetomaternal outcomes and psychophysical health in gestational diabetes mellitus. Biomedicine.

[19] Raison CL, Miller AH (2013). Role of inflammation in depression: implications for phenomenology, pathophysiology and treatment. Mod. Trends Pharmacopsych..

[20] Frank MS (2018). Serum markers of inflammation mediate the positive association between neuroticism and depression. Front. Psychiatry.

[21] Dowlati Y (2010). A meta-analysis of cytokines in major depression. Biol. Psychiatry.

[22] Khandaker GM, Pearson RM, Zammit S, Lewis G, Jones PB (2014). Association of serum interleukin 6 and C-reactive protein in childhood with depression and psychosis in young adult life: A population-based longitudinal study. JAMA Psych..

[23] Richardson AC, Carpenter MW (2007). Inflammatory mediators in gestational diabetes mellitus. Obstet. Gynecol. Clin. N. Am..

[24] Vrachnis N (2012). Role of adipokines and other inflammatory mediators in gestational diabetes mellitus and previous gestational diabetes mellitus. Int. J. Endocrinol..

[25] Robakis TK, Aasly L, Williams KE, Clark C, Rasgon N (2017). Roles of inflammation and depression in the development of gestational diabetes. Curr. Behav. Neurosci. Rep..

[26] Lappas M (2011). The Role of oxidative stress in the pathophysiology of gestational diabetes mellitus. Antioxid. Redox Signal..

[27] Lan Q (2022). Vascular endothelial dysfunction in gestational diabetes mellitus. Steroids.

[28] Jasmine MR, Nanda N, Sahoo J, Velkumary S, Pal GK (2020). Increased osteoprotegerin level is associated with impaired cardiovagal modulation in type-2 diabetic patients treated with oral antidiabetic drugs. BMC Cardiovasc. Disord..

[29] Auroprajna P (2018). Association of sympathovagal imbalance with cognitive impairment in type 2 diabetes in adults. Can. J. Diabetes.

[30] Indumathy J (2015). Decreased baroreflex sensitivity is linked to sympathovagal imbalance, body fat mass and altered cardiometabolic profile in pre-obesity and obesity. Metabolism.

[31] Kuppusamy S, Pal GK, Habeebullah S, Ananthanarayanan PH, Pal P (2015). Association of sympathovagal imbalance with cardiovascular risks in patients with polycystic ovary syndrome. Endocr. Res..

[32] Syamsunder AN (2013). Association of sympathovagal imbalance with cardiovascular risks in overt hypothyroidism. N. Am. J. Med. Sci..

[33] Pal GK, Shyma P, Habeebullah S, Shyjus P, Pal P (2009). Spectral analysis of heart rate variability for early prediction of pregnancy-induced hypertension. Clin. Exp. Hypertens..

[34] Pal GK (2011). Assessment of sympathovagal imbalance by spectral analysis of heart rate variability in prehypertensive and hypertensive patients in Indian population. Clin. Exp. Hypertens..

[35] Karthiga K (2021). Attenuation of baroreflex sensitivity and heart rate variability is linked to reduced levels of nitric oxide in pregnant women having risks of developing gestational hypertension. Clin. Exp. Hypertens..

[36] Rani PR, Begum J (2016). Screening and diagnosis of gestational diabetes mellitus, Where do we stand. J. Clin. Diagn. Res..

[37] Hayase M, Shimada M, Seki H (2014). Sleep quality and stress in women with pregnancy-induced hypertension and gestational diabetes mellitus. Women Birth.

[38] Seshiah V (2020). Diagnosis and principles of management of gestational diabetes mellitus in the prevailing COVID-19 pandemic. Int. J. Diab. Dev. Ctries..

[39] Balaji PA, Varne SR (2017). Physiological effects of yogasanas and pranayama on metabolic parameters, maternal and fetal outcome in gestational diabetes. Natl. J. Physiol. Pharm. Pharmacol..

[40] Meena R, Pal P, Papa D, Pal GK (2018). Increased rate pressure product is linked to sympathovagal imbalance in Indian obese postmenopausal women. Int. J. Clin. Exp. Physiol..

[41] Bosaeus M, Andersson-Hall U, Andersson L, Karlsson T, Ellegård L, Holmäng A (2020). Body composition during pregnancy: Longitudinal changes and method comparisons. Reprod. Sci..

[42] Obuchowska A, Standyło A, Kimber-Trojnar Ż, Leszczyńska-Gorzelak B (2021). The possibility of using bioelectrical impedance analysis in pregnant and postpartum women. Diagnostics (Basel).

[43] Task Force of the European Society of Cardiology and the North American Society of Pacing and Electrophysiology. Heart rate variability: Standards of measurement, physiological interpretation and clinical use. *Circulation* **93,** 1043–1065 (1996). 8598068

[44] Cohen S, Kamarck T, Mermelstein R (1983). A global measure of perceived stress. J. Health Soc. Behav..

[45] Cox JL, Holden JM, Sagovsky R (1987). Detection of postnatal depression. Development of the 10-item Edinburgh postnatal depression scale. Br. J. Psychiatry J. Ment. Sci..

[46] Flanagan JC (1978). A research approach to improving our quality of life. Am. Psychol..

[47] Hinkle S (2016). A longitudinal study of depression and gestational diabetes in pregnancy and the postpartum period. Diabetologia.

[48] Pace R, Rahme E, da Costa D, Dasgupta KK (2018). Association between gestational diabetes mellitus and depression in parents: A retrospective cohort study. Clin. Epidemiol..

[49] Dalfrà MG (2012). Quality of life in pregnancy and post-partum: A study in diabetic patients. Qual. Life Res..

[50] Haiying L, Jingsi C, Danxi Z (2008). Clinical investigation on mental health status of gestational diabetes patients. Matern. Child Health Care China.

[51] Lee KW (2019). Prevalence and factors associated with depressive, anxiety and stress symptoms among women with gestational diabetes mellitus in tertiary care centres in Malaysia: A cross-sectional study. BMC Preg. Childb..

[52] Dismuke CE, Hernandez-Tejada MA, Egede LE (2014). Relationship of serious psychological distress to quality of life in adults with diabetes. Int. J. Psychiatry Med..

[53] Trutnovsky G (2012). Gestational diabetes: Women's concerns, mood state, quality of life and treatment satisfaction. J. Matern. Fetal Neonatal Med..

[54] Ansarzadeh S, Salehi L, Mahmoodi Z, Mohammadbeigi A (2020). Factors affecting the quality of life in women with gestational diabetes mellitus: A path analysis model. Health Qual. Life Outcomes.

[55] Goldney RD, Phillips PJ, Fisher LJ, Wilson DH (2004). Diabetes, depression, and quality of life: A population study. Diab. Care.

[56] Martinelli LM (2008). Quality of life and its association with cardiovascular risk factors in a community health care program population. Clinics (Sao Paulo).

[57] Kassi E, Pervanidou P, Kaltsas G (2011). Metabolic syndrome: definitions and controversies. BMC Med..

[58] Patil R, Patil MSA (2021). Using Indian diabetes risk score to identify adult women at risk for diabetes. J. Food Nutr. Popul. Health.

[59] Mohan V (2007). Diabetes risk score helps identify metabolic syndrome and cardiovascular risk in Indians—The Chennai urban rural epidemiology study (CURES-38). Diab. Obes. Metab..

[60] Ting EY, Yang AC, Tsai SJ (2020). Role of interleukin-6 in depressive disorder. Int. J. Mol. Sci..

[61] García-Juárez M, Camacho-Morales A (2022). Defining the role of anti- and pro-inflammatory outcomes of interleukin-6 in mental health. Neuroscience.

[62] Zhao J, Shi W, Lu Y, Gao X, Wang A, Zhang S (2022). Alterations of monoamine neurotransmitters, HPA-axis hormones, and inflammation cytokines in reserpine-induced hyperalgesia and depression comorbidity rat model. BMC Psych..

[63] Anderson G (2013). Increased IL-6 trans-signaling in depression: Focus on the tryptophan catabolite pathway, melatonin and neuroprogression. Pharmacol. Rep..

[64] Wang R, Weng Y, Zhao S, Li S, Wen X, Huang G (2021). IL-6 up-regulates indoleamine 2, 3-dioxygenase (IDO) expression in chorionic villi and decidua. Xi Bao Yu Fen Zi Mian Yi Xue Za Zhi.

[65] Giacobbo BL (2019). Brain-derived neurotrophic factor in brain disorders: Focus on neuroinflammation. Mol. Neurobiol..

[66] Murthy KAS, Bhandiwada A, Chandan SL, Gowda SL, Sindhusree G (2018). Evaluation of oxidative stress and proinflammatory cytokines in gestational diabetes mellitus and their correlation with pregnancy outcome. Indian J. Endocrinol. Metab..

[67] Colm JM, Eszter T, Fergus PM, Cathal MM (2020). Mechanisms of endothelial dysfunction in pre-eclampsia and gestational diabetes mellitus: Windows into future cardiometabolic health?. Front. Endocrinol..

[68] Wareham NJ, Pfister R (2010). Diabetes: Glycated hemoglobin is a marker of diabetes and CVD risk. Nat. Rev. Cardiol..

[69] Horsch A (2016). Stress exposure and psychological stress responses are related to glucose concentrations during pregnancy. Br. J. Health Psychol..

[70] Pal GK, Pal P, Nanda N, Amudharaj D, Adithan C (2013). Cardiovascular dysfunctions and sympathovagal imbalance in hypertension and prehypertension: Physiological perspectives. Future Cardiol..

[71] Pal, G. K., Pal, P. & Nanda, N. Autonomic dysfunctions, autonomic function tests and spectral analysis of heart rate variability. in *Textbook of Medical Physiology*. (eds. Pal, G. K. & Pal, P.). 2nd edn. 214–222 (Elsevier, 2022).

[72] White WB (1999). Heart rate and the rate-pressure product as determinants of cardiovascular risk in patients with hypertension. Am. J. Hypertens..

[73] Bhavadharini B (2018). Elevated glycated hemoglobin predicts macrosomia among Asian Indian pregnant women (WINGS-9). Indian J. Endocrinol. Metab..

